# ﻿A new Asian leaf litter toad of the genus *Leptobrachella* (Amphibia, Anura, Megophryidae) from central south China

**DOI:** 10.3897/zookeys.1149.85895

**Published:** 2023-02-22

**Authors:** Jing Liu, Shengchao Shi, Shize Li, Mengfei Zhang, Sunjun Xiang, Gang Wei, Bin Wang

**Affiliations:** 1 Department of Resources and Environment, Moutai Institute, Renhuai 564500, China Department of Resources and Environment, Moutai Institute Renhuai China; 2 Chengdu Institute of Biology, Chinese Academy of Sciences, Chengdu 610041, China Chengdu Institute of Biology, Chinese Academy of Sciences Chengdu China; 3 Central South Inventory and Planning Institute of National Forestry and Grassland Administration, Changsha, Hunan 410014, China Central South Inventory and Planning Institute of National Forestry and Grassland Administration Changsha China; 4 College of Biological and Food Engineering, Huaihua University, Huaihua 418000, China Huaihua University Huaihua China; 5 Biodiversity Conservation Key Laboratory, Guiyang College, Guiyang 550002, Guizhou, China Guiyang College Guiyang China

**Keywords:** China, molecular phylogenetic analyses, morphology, new species, taxonomy

## Abstract

A new species of the Asian leaf litter toad genus *Leptobrachella* from central south China is described. Molecular phylogenetic analyses, based on mitochondrial 16S rRNA and nuclear RAG1 gene sequences indicated the new species as an independent clade in the genus. The new species could be distinguished from its congeners by a combination of the following characters: body of medium size (SVL 29.2–34.2 mm in 15 adult males and 34.4–43.1 mm in seven adult females); distinct black spots present on flanks; toes rudimentary webbed, with wide lateral fringes; ventral belly white with distinct nebulous brown speckling on ventrolateral flanks; skin on dorsum shagreened with fine tiny granules or short ridges; iris copper above, silver below; heels overlapped when thighs are positioned at right angles to the body; tibia-tarsal articulation reaches the middle eye; dorsal surface of tadpole semi-transparent light brown, spots on tail absent, keratodont row formula I: 3+3/2+2: I; call series basically consist of repeated long calls, at dominant frequency (5093 ± 412 Hz).

## ﻿Introduction

The Asian leaf litter toads of the genus *Leptobrachella* Smith, 1925 (Anura, Megophryidae) are widely distributed from southern China west to north-eastern India and Myanmar, through mainland Indochina to peninsular Malaysia and the island of Borneo (Chen et al. 2018; Frost 2022). The toads inhabit the forest floor in montane evergreen forest. The taxa in the group had been classified into different genera, i.e. *Paramegophrys* Liu, 1964, *Carpophrys* Sichuan Biological Research Institute, 1977, *Leptolalax* Dubois, 1980, *Lalax* Delorme, Dubois, Grosjean & Ohler, 2006 and *Lalos* Dubois, Grosjean, Ohler, Adler & Zhao, 2010. Based on large-scale molecular phylogenetic analyses on this group, Chen et al. (2018) suggested that the above genera were synonymised with *Leptobrachella*. Currently, the genus *Leptobrachella* contains 95 species, of which, as noted, 34 species were described in the last five years (Frost 2022). The species diversity in the genus was indicated to be much underestimated and many cryptic species have not been described until now (Chen et al. 2018).

In recent years, we carried out a series of biodiversity surveys in Hunan and Guizhou Provinces, China and collected some specimens of *Leptobrachella*. Morphological comparisons, molecular phylogenetic analyses and bioacoustic comparisons consistently indicated these specimens as an undescribed species. We describe it herein as a new species.

## ﻿Materials and methods

### ﻿Specimens

**Figure 1.**
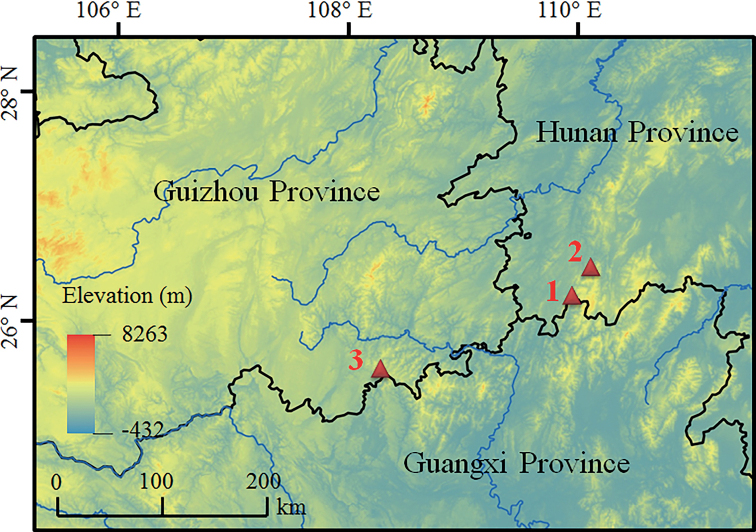
Sampling localities in this study. Localities 1–3 were all in China: 1, Tongdao County, Hunan Province; 2, Suining County, Hunan Province; 3, Congjiang County, Guizhou Province, China.

A total of 22 specimens of the new species (Suppl. material 1) were collected from Tongdao and Suining County, Hunan Province and Congjiang County, Guizhou Province, China (Fig. 1). After taking photographs, they were euthanised using isoflurane and then the specimens were fixed in 10% buffered formalin. Tissue samples were taken and preserved separately in 95% ethanol prior to fixation. Specimens were deposited in Chengdu Institute of Biology, the Chinese Academy of Sciences (**CIB**, **CAS**), China.

### ﻿Molecular phylogenetic analyses

A total of 15 samples of the new species were used for molecular analyses (Table 1). For phylogenetic analyses, the corresponding gene sequences for all those related species, for which comparable sequences were available, were also downloaded from GenBank (Table 1), based on previous studies (Chen et al. 2018; Shi et al. 2021). Corresponding sequences of one *Leptobrachiumhuashen* Fei & Ye, 2005 and one *Megophrysglandulosa* (Fei, Ye & Huang, 1990) were downloaded and used as outgroups.

**Table 1.** Information for samples used in molecular phylogenetic analyses in this study.

ID	Species	Locality	Voucher number	GenBank accession number	
				16S	RAG1
1	*Leptobrachelladong* sp. nov.	Tongdao County, Hunan Province, China	CIB SSC1758	OP764529	/
2	*Leptobrachelladong* sp. nov.	Tongdao County, Hunan Province, China	CIB SSC1757	OP764530	/
3	*Leptobrachelladong* sp. nov.	Tongdao County, Hunan Province, China	CIB WB2020277	OP764531	OP776448
4	*Leptobrachelladong* sp. nov.	Tongdao County, Hunan Province, China	CIB SSC1755	OP764532	/
5	*Leptobrachelladong* sp. nov.	Congjiang County, Guizhou Province, China	CIB LB20220305005	OP764533	/
6	*Leptobrachelladong* sp. nov.	Congjiang County, Guizhou Province, China	CIB LB20220306008	OP764534	OP776439
7	*Leptobrachelladong* sp. nov.	Congjiang County, Guizhou Province, China	CIB LB20220311002	OP764535	OP776441
8	*Leptobrachelladong* sp. nov.	Congjiang County, Guizhou Province, China	CIB LB20220311001	OP764536	OP776440
9	*Leptobrachelladong* sp. nov.	Congjiang County, Guizhou Province, China	CIB LB20220305001	OP764537	OP776449
10	*Leptobrachelladong* sp. nov.	Suining County, Hunan Province, China	CIB ZNY2022001	OP764538	OP776442
11	*Leptobrachelladong* sp. nov.	Suining County, Hunan Province, China	CIB ZNY2022002	OP764539	OP776443
12	*Leptobrachelladong* sp. nov.	Suining County, Hunan Province, China	CIB ZNY2022010	OP764540	OP776444
13	*Leptobrachelladong* sp. nov.	Suining County, Hunan Province, China	CIB ZNY2022011	OP764541	OP776445
14	*Leptobrachelladong* sp. nov.	Suining County, Hunan Province, China	CIB ZNY2022012	OP764542	OP776446
15	*Leptobrachelladong* sp. nov.	Suining County, Hunan Province, China	CIB ZNY2022013	OP764543	OP776447
16	*L.graminicola*	Mount Pu Ta Leng, Lao Cai, Vietnam	VNMN 010904	MZ224651	/
17	*L.graminicola*	Mount Pu Ta Leng, Lao Cai, Vietnam	VNMN 010905	MZ224648	/
18	*L.graminicola*	Mount Pu Ta Leng, Lao Cai, Vietnam	VNMN 010912	MZ224647	/
19	*L.graminicola*	Mount Pu Ta Leng, Lao Cai, Vietnam	VNMN 010908	MZ224653	/
20	*L.graminicola*	Mount Pu Ta Leng, Lao Cai, Vietnam	VNMN 010910	MZ224655	/
21	*L.graminicola*	Mount Pu Ta Leng, Lao Cai, Vietnam	VNMN 010909	MZ224649	/
22	*L.yeae*	Linggongli, Mount Emei, Sichuan Province, China	CIBEMLGL19052104	MT957006	MT975979
23	*L.yeae*	Mount Emei, Sichuan Province, China	SYS a001830	KM014810	/
24	*L.yeae*	Mount Emei, Sichuan Province, China	CIBEM1867	/	MT975978
25	*L.bourreti*	Mount Pu Ta Leng, Lao Cai, Vietnam	VNMN 010916	MZ209167	/
26	*L.bourreti*	Bat Xat District, Lao Cai, Vietnam	ZMMU-A5636-02280	MH055872	/
27	*L.bourreti*	Sapa, Lao Cai Province, Vietnam	AMS R 177673	KR018124	/
28	*L.bourreti*	Ky Quan San, Lao Cai, Vietnam	AMS R 188515	MZ208835	/
29	*L.bourreti*	Sapa, Lao Cai, Vietnam	1999.566	KR827860	/
30	*L.chishuiensis*	Chishui National Nature Reserve, Chishui City, Guizhou Province, China	CIBCS20190518042	MT117054	/
31	*L.cf.oshanensis*	Changning County, Sichuan Province, China	CIB20050095	KC460337	/
32	*L.bijie*	Zhaozishan Nature Reserve, Bijie City, Guizhou Province, China	SYS a007314	MK414533	/
33	*L.jinshaensis*	Lengshuihe Nature Reserve, Jinsha County, Guizhou Province, China	CIB JS20200516001	MT814014	/
34	*L.suiyangensis*	Huoqiuba Nature Reserve, Suiyang County, Guizhou Province, China	GZNU20180606005	MK829649	OL800396
35	*L.suiyangensis*	Huoqiuba Nature Reserve, Suiyang County, Guizhou Province, China	GZNU20180606002	/	OL800395
36	*L.purpuraventra*	Wujing Nature Reserve, Bijie City, Guizhou Province, China	SYS a007081	MK414517	/
37	*L.niveimontis*	Daxueshan Nature Reserve, Yunnan Province, China	KIZ015734	MT302618	/
38	*L.wulingensis*	Tianquanshan Forest Park, Zhangjiajie, Hunan Province, China	CSUFT 177	MT530315	/
39	*L.cf.oshanensis*	Nanchuan District, Chongqing City, China	ZYC799	AY526215	/
40	*L.dorsospina*	Yushe Forest Park, Shuicheng County, Guizhou Province, China	SYS a004961	MW046194	/
41	*L.alpina*	Caiyanghe, Yunnan Province, China	KIZ049024	MH055867	MH056093
42	*L.purpura*	Yingjiang, Yunnan Province, China	SYS a006530	MG520354	/
43	*L.eos*	Boun Tay, Phongsaly, Laos	NCSM 80551	MH055887	/
44	*L.oshanensis*	Baoguosi, Mount Emei, Sichuan Province, China	CIBEMS20190421BGS1	MT957023	MT975988
45	*L.oshanensis*	Shengshuige, Mount Emei, Sichuan Province, China	CIBEMS20190422SSG1-4		MT975985
46	*L.murphyi*	Doi Inthanon, Chiang Mai, Thailand	KIZ034039	MZ710519	/
47	*L.yunyangensis*	Qiyaoshan Nature Reserve, Yunyang County, Chongqing, China	GZNU20210622001	OL800364	OL800393
48	*L.yunyangensis*	Qiyaoshan Nature Reserve, Yunyang County, Chongqing, China	GZNU20210622002	/	OL800394
49	*L.tengchongensis*	Gaoligong Shan, Yunnan Province, China	SYS a004598	KU589209	/
50	*L.khasiorum*	Khasi Hills, Meghalaya, India	SDBDU 2009.329	KY022303	KY022348
51	*L.tamdil*	Mizoram, India	MZMU2224	MW665130	/
52	*L.yingjiangensis*	Yingjiang County, Yunnan Province, China	SYS a006533	MG520350	/
53	*L.petrops*	Ba Vi National Park, Ha Tay, Vietnam	ROM 13483	MH055901	MH056092
54	*L.puhoatensis*	Pu Hu, Thanh Hoa, Vietnam	VNMN:2016 A.23	KY849587	/
55	*L.namdongensis*	Thanh Hoa Province, Vietnam	VNUF A.2017.37	MK965389	/
56	*L.liui*	Wuyi Shan City, Fujian Province, China	SYS a001597	KM014547	/
57	*L.mangshanensis*	Mangshan, Hunan Province, China	MSZTC201701	MG132196	/
58	*L.verrucosa*	Lianshan Bijiashan Nature Reserve, Guangdong, China	GEP a059	OP279589	/
59	*L.shimentaina*	Shimentai Nature Reserve, Guangdong, China	SYS a004712	MH055926	/
60	*L.bashaensis*	Basha Nature Reserve, Congjiang County, Guizhou Province, China	GIB196403	MW136294	/
61	*L.maoershanensis*	Mao’er Shan, Guangxi Province, China	KIZ07614	MH055927	MH056099
62	*L.laui*	Shenzhen City, Guangdong Province, China	SYS a002450	MH055904	/
63	*L.yunkaiensis*	Dawuling Forest Station, Maoming City, Guangdong Province, China	SYS a004663	MH605584	/
64	*L.flaviglandulosa*	Xiaoqiaogou Nature Reserve, Yunnan Province, China	KIZ016072	MH055934	MH056098
65	*L.firthi*	Quang Nam Province, Vietnam	AMS R 171714	JQ739203	/
66	*L.isos*	Gia Lai, Vietnam	AMS R 176469	KT824767	/
67	*L.sungi*	Tam Dao, Vinh Phuc, Vietnam	ROM 20236	MH055858	MH056104
68	*L.zhangyapingi*	Chiang Mai, Thailand	KIZ07258	MH055864	MH056102
69	*L.aspera*	Huanglianshan Nature Reserve, Lyuchun, Yunnan, China	SYS a007743	MW046199	/
70	*L.feii*	Xiaoqiaogou Nature Reserve, Yunnan Province, China	KIZ048894	MT302634	/
71	*L.pelodytoides*	Tam Dao, Vinh Phu, Vietnam	ROM18282	EF397244	/
72	*L.ventripunctata*	Wenlong, Yunnan Province, China	KIZ013621	MH055824	MH056090
73	*L.aerea*	Vilabuly, Savannakhet, Laos	NCSM 76038	MH055809	/
74	*L.minima*	Doi Phu Fa, Nan, Thailand	KIZ024317	MH055852	MH056091
75	*L.shiwandashanensis*	Fangcheng City, Guangxi Province, China	NNU202103146	MZ326691	/
76	*L.wuhuangmontis*	Pubei County, Guangxi Province, China	SYS a003485	MH605577	/
77	*L.damingshanensis*	Guangxi Province, China	NNU202103281	MZ145229	/
78	*L.nyx*	Ha Giang, Vietnam	ROM 36692	MH055816	/
79	*L.nahangensis*	Na Hang Nature Reserve, Tuyen Quang, Vietnam	ROM 7035	MH055853	/
80	*L.pluvialis*	Fansipan, Lao Cai, Vietnam	ROM 30685	MH055843	/
81	*L.shangsiensis*	Guangxi Province, China	NHMG1401032	MK095460	/
82	*L.pallida*	Lam Dong, Vietnam	UNS00511	KU530190	/
83	*L.kalonensis*	Binh Thuan Province, Vietnam	AMNH A191762	KR018115	/
84	*L.bidoupensis*	Bidoup-Nui Ba National Park, Lam Dong, Vietnam	ZMMU-A-4797-01454	MH055945	MH056110
85	*L.tadungensis*	Dak Nong Province, Vietnam	UNS00515	KR018121	/
86	*L.maculosa*	Ninh Thuan Province, Vietnam	AMS R 177660	KR018119	/
87	*L.pyrrhops*	Loc Bac, Lam Dong, Vietnam	ZMMU-A-4873-00158	MH055950	MH056109
88	*L.macrops*	Phu Yen, Vietnam	ZMMU-A5823	MG787993	/
89	*L.melica*	Ratanakiri, Cambodia	MVZ258198	HM133600	/
90	*L.rowleyae*	Da Nang City, Vietnam	ITBCZ2783	MG682552	/
91	*L.applebyi*	Phong Dien Nature Reserve, Thua Thien-Hue, Vietnam	KIZ010701	MH055947	MH056105
92	*L.ardens*	Kon Ka Kinh National Park, Gia Lai, Vietnam	ZMMU-NAP-06099	MH055949	MH056108
93	*L.crocea*	Thua Thien-Hue, Vietnam	ZMMU-NAP-02274	MH055955	MH056114
94	*L.tuberosa*	Kon Ka Kinh National Park, Gia Lai, Vietnam	ZMMU-NAP-02275	MH055959	MH056111
95	*L.botsfordi*	Fansipan, Lao Cai, Vietnam	AMS R 176540	MH055952	MH056088
96	*L.fuliginosa*	Phetchaburi, Thailand	KUHE 20197	LC201988	/
97	*L.melanoleuca*	Kapoe, Ranong, Thailand	KIZ018031	MH055967	MH056115
98	*L.neangi*	Veal Veng District, Pursat Province, Cambodia	CBC 1624	MT644613	/
99	*L.heteropus*	Larut, Perak, Malaysia	KUHE15487	AB530453	/
100	*L.sola*	Gunung Stong, Kelantan, Malaysia	KU RMB20973	MH055973	MH056119
101	*L.kecil*	Cameron, Malaysia	KUHE 52440	LC202004	/
102	*L.kajangensis*	Tioman, Malaysia	LSUHC 4431	LC202001	/
103	*L.dringi*	Gunung Mulu, Malaysia	KUHE:55610	AB847553	/
104	*L.sabahmontana*	Borneo, Malaysia	BORNEENSIS 12632	AB847551	/
105	*L.picta*	Borneo, Malaysia	UNIMAS 8705	KJ831295	/
106	*L.fritinniens*	Danum Valley Field Center, Sabah, Malaysia	FMNH 244800	MH055971	MH056118
107	*L.arayai*	Liwagu, Kinabalu, Malaysia	BORNEEISIS 22931	AB847558	/
108	*L.hamidi*	Bukit Lanjan, Selangor, Malaysia	KUHE17545	AB969286	/
109	*L.marmorata*	Borneo, Malaysia	KUHE53227	AB969289	/
110	*L.gracilis*	Bukit Kana, Sarawak, Malaysia	FMNH 273682	MH055972	MH056117
111	*L.maura*	Borneo, Malaysia	SP 21450	AB847559	/
112	*L.baluensis*	Tambunan, Sabah, Borneo, Malaysia	SP 21604	LC056792	/
113	*L.parva*	Mulu National Park, Sarawak, Malaysia	KUHE:55308	LC056791	MH056121
114	*L.brevicrus*	Gunung Mulu National Park, Sarawak, Malaysia	UNIMAS 8957	KJ831303	/
115	*L.itiokai*	Mulu National Park, Sarawak, Malaysia	KUHE:5589	LC137805	MH056120
116	*L.mjobergi*	Gading NP, Sarawak, Borneo, Malaysia	KUHE:47872	LC056787	/
117	*L.juliandringi*	Mulu NP, Sarawak, Borneo, Malaysia	KUHE 55333	LC056780	/
118	*Megophrysglandulosa*	Yunnan Province, China	KIZ048439	KX811762	MH056125
119	*Leptobrachiumhuashen*	Yunnan Province, China	KIZ049025	KX811931	MH056122

Total DNA was extracted using a standard phenol-chloroform extraction protocol (Sambrook et al. 1989). The mitochondrial 16S rRNA gene and nuclear DNA recombination activating gene 1 (RAG1) were amplified and the primers P7 (5’-CGCCTGTTTACCAAAAACAT-3’) and P8 (5’-CCGGTCTGAACTCAGATCACGT-3’) for 16S were used following Simon et al. (1994) and RAG1_F (5’-AGCTGCAGYCARTACCAYAARATGTA-3’) and RAG1_R (5’-GCAAAGTTTCCGTTCATTCTCAT-3’) for RAG1 were used following Mauro et al. (2004). Gene fragments were amplified under the following conditions: an initial denaturing step at 95 °C for 4 min; 36 cycles of denaturing at 95 °C for 30 sec, annealing at 51 °C/54 °C (16S/ RAG1) for 30 sec and extension at 72 °C for 70 sec, followed by a final extending step at 72 °C for 10 min. The fragments were sequenced on an ABI Prism 3730 automated DNA sequencer (Applied Biosystems, USA). New sequences were deposited in GenBank (for GenBank accession numbers, see Table 1).

Sequences were assembled and aligned using the Clustalw module in BioEdit v. 7.0.9.0 (Hall 1999) with default settings. Alignments were checked by eye and revised manually, if necessary. For the phylogenetic analyses, we constructed two sequence matrices for reconstructing the phylogenetic trees, i.e. mitochondrial 16S and RAG1 gene datasets. Phylogenetic analyses were conducted using Maximum Likelihood (ML) and Bayesian Inference (BI) methods, implemented in PhyML v. 3.0 (Guindon et al. 2010) and MrBayes v. 3.12 (Ronquist and Huelsenbeck 2003), respectively. We ran jModelTest v. 2.1.2 (Darriba et al. 2012) with Akaike and Bayesian Information Criteria on the alignment, resulting in the best-fitting nucleotide substitution models of GTR + I + G for the 16S data and GTR + R for the RAG1 data. For the ML tree, branch supports were drawn from 10,000 non-parametric bootstrap replicates. In BI analyses, the parameters for each partition were unlinked and branch lengths were allowed to vary proportionately across partitions. Two runs each with four Markov chains were simultaneously run for 60 million generations with sampling every 1,000 generations. The first 25% trees were removed as the “burn-in” stage followed by calculations of Bayesian posterior probabilities and the 50% majority-rule consensus of the post burn-in trees sampled at stationarity. To detect the haplotype relationships and genetic isolation between the undescribed species and its related species on nuclear DNA, a haplotype network, based on RAG1 gene sequences, was constructed using the maximum parsimony method in TCS v.1.21 (Clement et al. 2000). Finally, genetic distance between *Leptobrachella* species, based on uncorrected *p*-distance model, was estimated on 16S gene using MEGA v.6.06 (Tamura et al. 2013).

### ﻿Morphological comparisons

All 22 specimens of the new taxon were measured. The terminology and methods followed Fei and Ye (2005), Mahony et al. (2011), Wang et al. (2019) and Shi et al. (2021). Measurements were made with a dial caliper to the nearest 0.1 mm (Watters et al. 2016) with digital calipers. Fourteen morphometric characters of adult specimens were measured:

**ED** eye diameter (distance from the anterior corner to the posterior corner of the eye);

**FL** foot length (distance from tarsus to the tip of the fourth toe);

**HDL** head length (distance from the tip of the snout to the articulation of jaw);

**HDW** head width (greatest width between the left and right articulations of jaw);

**HLL** hind-limb length (distance from tip of fourth toe to vent);

**IND** internasal distance (minimum distance between the inner margins of the external nares);

**IOD** interorbital distance (minimum distance between the inner edges of the upper eyelids);

**LAL** length of lower arm and hand (distance from the elbow to the distal end of the Finger IV);

**ML** manus length (distance from tip of third digit to proximal edge of inner palmar tubercle);

**SL** snout length (distance from the tip of the snout to the anterior corner of the eye);

**TL** tibia length (distance from knee to tarsus);

**TYD** maximal tympanum diameter;

**UEW** upper eyelid width (greatest width of the upper eyelid margins measured perpendicular to the anterior-posterior axis).

One tadpole specimen of the undescribed species was measured. Nineteen morphometric characters were measured for the tadpole:

**BH** maximum body height;

**BL** body length, from tip of snout to conjunction of body and tail;

**BW** maximum body width;

**ED** eye diameter (distance from the anterior corner to the posterior corner of the eye);

**IND** internasal distance (minimum distance between the inner margins of the external nares);

**KRF** keratodont row formula;

**LF** maximum height of lower tail fin;

**NE** distance between nostril and eye;

**ODW** oral disc width;

**PP** interpupilar distance;

**RN** rostro-narial distance;

**SN** snout length, from tip of snout to the anterior corner of eye;

**SS** distance from tip of snout to opening of spiracle;

**SU** distance from snout to beginning of upper tail fin;

**TAL** tail length;

**TMH** maximum tail muscle height;

**TMW** maximum tail muscle width;

**UF** maximum height of upper tail fin;

**TH** maximum tail height.

The new taxon was also compared with all other congeners of *Leptobrachella*, based on morphological characters. Comparative morphological data were obtained from literature (Table 2).

**Table 2.** References for morphological characters for congeners of the genus *Leptobrachella*.

ID	Species	Literature reviewed
1	*L.aerea* (Rowley, Stuart, Richards, Phimmachak & Sivongxay, 2010)	Rowley et al. (2010c)
2	*L.alpina* (Fei, Ye & Li, 1990)	Fei et al. (2009)
3	*L.applebyi* (Rowley & Cao, 2009)	Rowley and Cao (2009)
4	*L.arayai* (Matsui, 1997)	Matsui (1997)
5	*L.ardens* (Rowley, Tran, Le, Dau, Peloso, Nguyen, Hoang, Nguyen & Ziegler, 2016)	Rowley et al. (2016)
6	*L.aspera* Wang, Lyu, Qi & Wang, 2020	Wang et al. (2020)
7	*L.baluensis* Smith, 1931	Dring (1983); Eto et al. (2016)
8	*L.bashaensis* Lyu, Dai, Wei, He, Yuan, Shi, Zhou, Ean, Kuang, Guo, Wei & Yuan, 2020	Lyu et al. (2020)
9	*L.bidoupensis* (Rowley, Le, Tran & Hoang, 2011)	Rowley et al. (2011)
10	*L.bijie* Wang, Li, Li, Chen & Wang, 2019	Wang et al. (2019)
11	*L.bondangensis* Eto, Matsui, Hamidy, Munir & Iskandar, 2018	Eto et al. (2018)
12	*L.botsfordi* (Rowley, Dau & Nguyen, 2013)	Rowley et al. (2013)
13	*L.bourreti* (Dubois, 1983)	Ohler et al. (2011); Nguyen et al. (2021)
14	*L.brevicrus* Dring, 1983	Dring (1983); Eto et al. (2015)
15	*L.chishuiensis* (Li, Liu, Wei & Wang, 2020)	Li et al. (2020)
16	*L.crocea* (Rowley, Hoang, Le, Dau & Cao, 2010)	Rowley et al. (2010a)
17	*L.damingshanensis* Chen, Yu, Cheng, Meng, Wei, Zhou & Lu, 2021	Chen et al. (2021b)
18	*L.dorsospina* Wang, Lyu, Qi & Wang, 2020	Wang et al. (2020)
19	*L.dringi* (Dubois, 1987)	Inger et al. (1995); Matsui and Dehling (2012)
20	*L.eos* (Ohler, Wollenberg, Grosjean, Hendrix, Vences, Ziegler & Dubois, 2011)	Ohler et al. (2011)
21	*L.feii* (Chen, Yuan & Che, 2020)	Chen et al. (2020)
22	*L.firthi* (Rowley, Hoang, Dau, Le & Cao, 2012)	Rowley et al. (2012)
23	*L.flaviglandulosa* (Chen, Wang & Che, 2020)	Chen et al. (2020)
24	*L.fritinniens* (Dehling & Matsui, 2013)	Dehling and Matsui (2013)
25	*L.fuliginosa* (Matsui, 2006)	Matsui (2006)
26	*L.fusca* Eto, Matsui, Hamidy, Munir & Iskandar, 2018	Eto et al. (2018)
27	*L.gracilis* (Günther, 1872)	Günther (1872); Dehling (2012b)
28	*L.graminicola* Nguyen, Tapley, Nguyen, Luong & Rowley, 2021	Nguyen et al. (2021)
29	*L.hamidi* (Matsui, 1997)	Matsui (1997)
30	*L.heteropus* (Boulenger, 1900)	Boulenger (1900)
31	*L.isos* (Rowley, Stuart, Neang, Hoang, Dau, Nguyen & Emmett, 2015)	Rowley et al. (2015a)
32	*L.itiokai* Eto, Matsui & Nishikawa, 2016	Eto et al. (2016)
33	*L.jinshaensis* Cheng, Shi, Li, Liu, Li & Wang, 2021	Cheng et al. (2021)
34	*L.juliandringi* Eto, Matsui & Nishikawa, 2015	Eto et al. (2015)
35	*L.kajangensis* (Grismer, Grismer & Youmans, 2004)	Grismer et al. (2004)
36	*L.kalonensis* (Rowley, Tran, Le, Dau, Peloso, Nguyen, Hoang, Nguyen & Ziegler, 2016)	Rowley et al. (2016)
37	*L.kecil* (Matsui, Belabut, Ahmad & Yong, 2009)	Matsui et al. (2009)
38	*L.khasiorum* (Das, Tron, Rangad & Hooroo, 2010)	Das et al. (2010)
39	*L.lateralis* (Anderson, 1871)	Anderson (1871); Humtsoe et al. (2008)
40	*L.laui* (Sung, Yang & Wang, 2014)	Sung et al. (2014)
41	*L.liui* (Fei & Ye, 1990)	Fei et al. (2009); Sung et al. (2014)
42	*L.macrops* (Duong, Do, Ngo, Nguyen & Poyarkov, 2018)	Duong et al. (2018)
43	*L.maculosa* (Rowley, Tran, Le, Dau, Peloso, Nguyen, Hoang, Nguyen & Ziegler, 2016)	Rowley et al. (2016)
44	*L.mangshanensis* (Hou, Zhang, Hu, Li, Shi, Chen, Mo & Wang, 2018)	Hou et al. (2018)
45	*L.maoershanensis* (Yuan, Sun, Chen, Rowley & Che, 2017)	Yuan et al. (2017)
46	*L.marmorata* (Matsui, Zainudin & Nishikawa, 2014)	Matsui et al. (2014b)
47	*L.maura* (Inger, Lakim, Biun & Yambun, 1997)	Inger et al. (1997)
48	*L.melanoleuca* (Matsui, 2006)	Matsui (2006)
49	*L.melica* (Rowley, Stuart, Neang & Emmett, 2010)	Rowley et al. (2010b)
50	*L.minima* (Taylor, 1962)	Taylor (1962); Ohler et al. (2011)
51	*L.mjobergi* (Smith, 1925)	Eto et al. (2015)
52	*L.murphyi* Chen, Suwannapoom, Wu, Poyarkov, Xu, Pawangkhanant & Che, 2021	Chen et al. (2021a)
53	*L.namdongensis* (Hoang, Nguyen, Luu, Nguyen & Jiang, 2019)	Hoang et al. (2019)
54	*L.nahangensis* (Lathrop, Murphy, Orlov & Ho, 1998)	Lathrop et al. (1998)
55	*L.natunae* (Günther, 1895)	Günther (1895)
56	*L.neangi* Stuart & Rowley, 2020	Stuart and Rowley (2020)
57	*L.niveimontis* (Chen, Poyarkov, Yuan & Che, 2020)	Chen et al. (2020)
58	*L.nokrekensis* (Mathew & Sen, 2010)	Mathew and Sen (2010)
59	*L.nyx* (Ohler, Wollenberg, Grosjean, Hendrix, Vences, Ziegler & Dubois, 2011)	Ohler et al. (2011)
60	*L.oshanensis* (Liu, 1950)	Fei et al. (2009)
61	*L.pallida* (Rowley, Tran, Le, Dau, Peloso, Nguyen, Hoang, Nguyen & Ziegler, 2016)	Rowley et al. (2016)
62	*L.palmata* Inger & Stuebing, 1992	Inger and Stuebing (1992)
63	*L.parva* Dring, 1983	Dring (1983)
64	*L.pelodytoides* (Boulenger, 1893)	Boulenger (1893); Ohler et al. (2011)
65	*L.petrops* (Rowley, Dau, Hoang, Le, Cutajar & Nguyen, 2017)	Rowley et al. (2017a)
66	*L.picta* (Malkmus, 1992)	Malkmus (1992)
67	*L.pingbianensis* (Rao, Hui, Zhu & Ma, 2022)	Rao (2022 “2020”)
68	*L.platycephala* (Dehling, 2012)	Dehling (2012a)
69	*L.pluvialis* (Ohler, Marquis, Swan & Grosjean, 2000)	Ohler et al. (2000, 2011)
70	*L.puhoatensis* (Rowley, Dau & Cao, 2017)	Rowley et al. (2017b)
71	*L.purpuraventra* Wang, Li, Li, Chen & Wang, 2019	Wang et al. (2019)
72	*L.purpurus* (Yang, Zeng & Wang, 2018)	Yang et al. (2018)
73	*L.pyrrhops* (Poyarkov, Rowley, Gogoleva, Vassilieva, Galoyan & Orlov, 2015)	Poyarkov et al. (2015)
74	*L.rowleyae* (Nguyen, Poyarkov, Le, Vo, Ninh, Duong, Murphy & Sang, 2018)	Nguyen et al. (2018)
75	*L.sabahmontana* (Matsui, Nishikawa & Yambun, 2014)	Matsui et al. (2014a)
76	*L.serasanae* Dring, 1983	Dring (1983)
77	*L.shangsiensis* Chen, Liao, Zhou & Mo, 2019	Chen et al. (2019)
78	*L.shimentaina* Wang, Lyu & Wang, 2022	Wang et al. (2022)
79	*L.shiwandashanensis* Chen, Peng, Pan, Liao, Liu & Huang, 2021	Chen et al. (2021c)
80	*L.sola* (Matsui, 2006)	Matsui (2006)
81	*L.sungi* (Lathrop, Murphy, Orlov & Ho, 1998)	Lathrop et al. (1998)
82	*L.suiyangensis* (Luo, Xiao, Gao & Zhou, 2020)	Luo et al. (2020)
83	*L.tadungensis* (Rowley, Tran, Le, Dau, Peloso, Nguyen, Hoang, Nguyen & Ziegler, 2016)	Rowley et al. (2016)
84	*L.tamdil* (Sengupta, Sailo, Lalremsanga, Das & Das, 2010)	Sengupta et al. (2010)
85	*L.tengchongensis* (Yang, Wang, Chen & Rao, 2016)	Yang et al. (2016)
86	*L.tuberosa* (Inger, Orlov & Darevsky, 1999)	Inger et al. (1999)
87	*L.ventripunctata* (Fei, Ye & Li, 1990)	Fei et al. (2009)
88	*L.verrucosa* Wang, Zeng, Lin & Li, 2022	Lin et al. (2022)
89	*L.wuhuangmontis* Wang, Yang & Wang, 2018	Wang et al. (2018)
90	*L.wulingensis* Qian, Xiao, Cao, Xiao & Yang, 2020	Qian et al. (2020)
91	*L.yeae* Shi, Hou, Song, Jiang & Wang, 2021	Shi et al. (2021)
92	*L.yingjiangensis* (Yang, Zeng & Wang, 2018)	Yang et al. (2018)
93	*L.yunkaiensis* Wang, Li, Lyu & Wang, 2018	Wang et al. (2018)
94	*L.yunyangensis* Luo, Deng & Zhou, 2022	Luo et al. (2022)
95	*L.zhangyapingi* (Jiang, Yan, Suwannapoom, Chomdej & Che, 2013)	Jiang et al. (2013)

### ﻿Bioacoustics analyses

The advertisement calls of the new taxon were recorded from specimens CIB SSC1757, CIB SSC1754, CIB SSC1760, CIB LB20220311001, CIB LB20220311002 and CIB ZNY2022012. The advertisement call of the toad was recorded in the stream at ambient air temperature of 11.2–15.0 °C. Sony PCM-D50, Philips VTR 6900 digital sound recorder, Huawei Mate 30E Pro smart phone were used to record within 20 cm of the calling individual. The sound files in wave format were resampled at 48 kHz with sampling depth 24 bits. Terminology of advertisement call analyses and description followed Köhler et al. (2017) and Wang et al. (2019). Call recordings were analysed and visualised by Raven Pro 1.5 software (Cornell Laboratory of Ornithology, Ithaca, NY, USA) (window size 256 points, fast-Fourier transform, Hanning windows). Ambient temperature was taken by a digital hygrothermograph.

## ﻿Results

**Figure 2.**
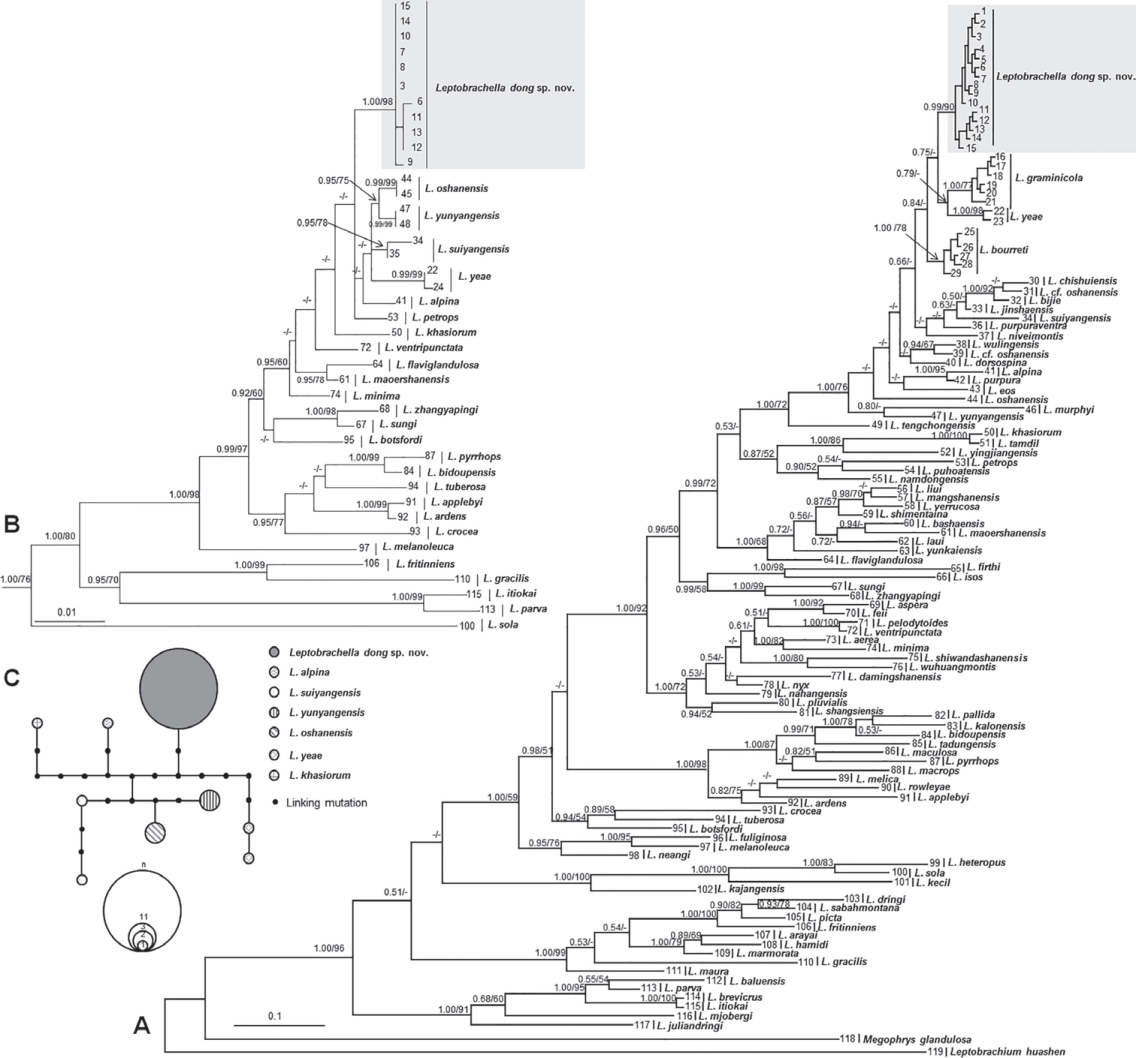
Phylogenetic trees of the genus *Leptobrachella* and a haplotype network constructed based on RAG1 gene sequences **A** maximum Likelihood (ML) tree reconstructed, based on mitochondrial 16S gene sequences **B** maximum Likelihood tree reconstructed, based on nuclear RAG1 gene sequences. Bayesian posterior probabilities (BPP) from BI analyses/ bootstrap supports (BS) from ML analyses are listed beside the nodes. The symbol “-” represents a value below 0.50/50. For information of samples 1–119, refer to Table 1**C** the haplotype network constructed, based on RAG1 gene sequences.

The aligned sequence matrix of 16S and RAG1 gene contained 498 bps and 888 bps, respectively. ML and BI analyses, based on the 16S gene matrix, resulted in essentially identical topologies (Fig. 2). All 15 samples of the undescribed species were clustered into one clade being deeply clustered into the *Leptobrachella* clade and seemly being sister to a clade comprising of *L.graminicola* and *L.yeae* (Fig. 2A). ML and BI analyses, based on the RAG1 gene matrix, also resulted in essentially identical topologies (Fig. 2B). In the RAG1 tree, eleven samples of the undescribed species were clustered together into an independent clade which was distantly divergent from other congeners. Four haplotypes were found for eleven samples of the undescribed species in the RAG1 gene and there was no common haplotype between the undescribed species and its related species (Fig. 2C). The genetic distance of the *p*-distance model on the 16S gene within the specimens of the undescribed species ranged from 0.0% to 0.4%, being much lower than the interspecific genetic distance (all higher than 1.8%; Suppl. material 2). The smallest pairwise genetic divergence between the undescribed species and its congeners is 2.2% (vs. *L.bourreti*), being higher than or at the same level with that between some pairs of related species, such as *L.bijie* and *L.jinshaensis* (2.0%), *L.purpuraventra* and and *L.jinshaensis* (2.1%).

The undescribed species could be identified from its congeners in a series of morphological and bioacoustics characters. For the detailed demonstration, based on morphological and bioacoustics comparisons, see the following section describing the new species.

Molecular phylogenetic analyses, morphological comparisons and bioacoustics analyses indicated that the specimens from Tongdao and Suining County, Hunan Province and Congjiang County, Guizhou Province, China represent an undescribed species which is described as follows.

### ﻿Taxonomic account

#### ﻿ Leptobrachella dong sp. nov.

Taxon classificationAnimaliaAnuraMegophryidae

1FBBEDDB-99F0-5DBF-9645-B8542C4D0F24

https://zoobank.org/EECEF9D0-E00F-49E3-B576-8E3A11C688DB

**Figure 3.**
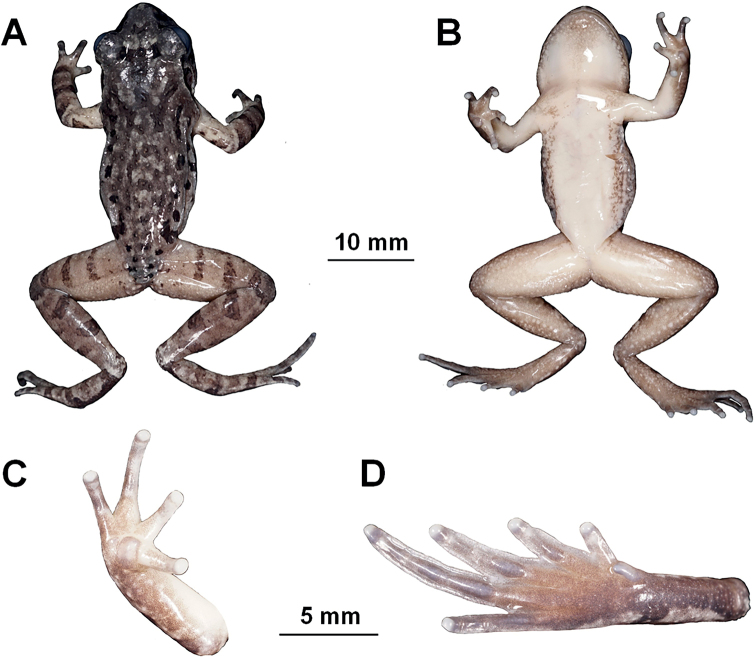
The holotype specimen CIB SSC1757 of *Leptobrachelladong* sp. nov. **A** dorsal view **B** ventral view **C** ventral view of hand **D** ventral view of foot.

**Figure 4.**
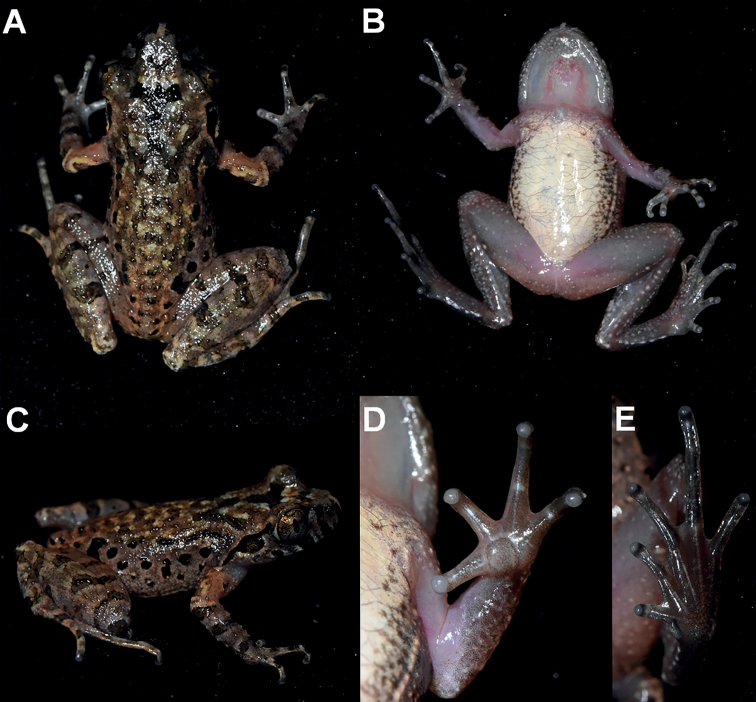
Photos of the holotype CIB SSC1757 of *Leptobrachelladong* sp. nov. in life **A** dorsal view **B** ventral view **C** dorsolateral view **D** ventral view of hand **E** ventral view of foot.

**Figure 5.**
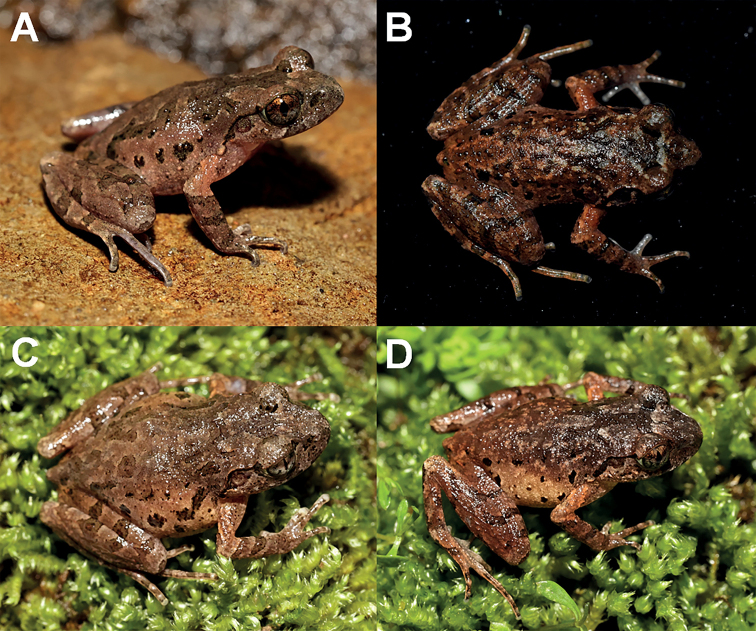
Colour variation in *Leptobrachelladong* sp. nov. **A** dorsolateral view of the male specimen CIB LB20220311002 **B** dorsal view of the male specimen CIB SCC1754 **C** dorsal view of the female specimen CIB LB20220305005 **D** dorsal view of the female specimen CIB LB20220306008.

Figs 3 , 4 , 5 ; Tables 1 , 2 , 3 , 4 ; Suppl. materials 1 , 2 , 3

##### Type materials.

***Holotype*.**CIB SSC1757, adult male (Figs 3, 4), collected by Shengchao Shi in Tongdao County (26.206674°N, 109.952695°E, ca. 790 m a.s.l.), Hunan Province, China on 2 April 2017.

***Paratypes*.** Four adult males CIB SSC1754, CIB SSC1758, CIB SSC1759, CIB SSC1760, one adult female CIB SSC1755 and one tadpole CIB WB2020277 from the same place as holotype collected by Sheng-Chao Shi. Two adult males CIB LB20220305001 and CIB LB20220305003 and five adult females CIB LB20220306003, CIB LB20220306005, CIB LB20220305010, CIB LB20220306008 and CIB LB20220306009 collected by Shize Li from Congjiang County (25.572492°N, 108.274189°E, 1200 m a.s.l.), Guizhou Province, China on 6 March 2022. Two adult males CIB LB20220311001 and CIB LB20220311002 collected by Jing Liu from the same place as Congjiang County, Guizhou Province, China on 11 March 2022; One adult female CIB ZNY2022003 and two adult males CIB ZNY2022001 and CIB ZNY2022002 collected by Fu Shu and four adult males CIB ZNY2022010, CIB ZNY2022011, CIB ZNY2022012 and CIB ZNY2022013 collected by Keji Guo from the same place as Suining County (26.401561°N, 110.093467°E, 620 m a.s.l.), Hunan Province, China on 15 March 2022.

##### Diagnosis.

*Leptobrachelladong* sp. nov. is assigned to the genus *Leptobrachella*, based on molecular data and the following morphological characters: medium size, rounded finger tips, the presence of an elevated inner palmar tubercle not continuous to the thumb, presence of macroglands on body (including supra-axillary, pectoral and femoral glands), vomerine teeth absent, tubercles on eyelids and anterior tip of snout with vertical white bar (Dubois 1983; Fei et al. 2009).

*Leptobrachelladong* sp. nov. could be distinguished from its congeners by a combination of the following characters: (1) body of medium size (SVL 29.2–32.0 mm in 15 adult males and 37.4–43.1 mm in seven adult females); (2) distinct black spots present on flanks; toes rudimentary webbed, with wide lateral fringes; (3) ventral belly white with distinct nebulous brown speckling on ventrolateral flanks; (4) skin on dorsum shagreened with fine tiny granules or short ridges; (5) heels overlapped when thighs are positioned at right angles to the body; (6) tibia-tarsal articulation reaches the middle eye; (7) dorsal surface of tadpole semi-transparent light brown, spots on tail absent, keratodont row formula I: 3+3/2+2: I; (8) calls with two types, at dominant frequency (5.1 ± 0.4 kHz).

##### Description of holotype.

Adult male. SVL in 32.0 mm. Head width almost equal with head length slightly (HDW / HDL 1.03); snout rounded in both ventral view and lateral view, projecting slightly beyond margin of the lower jaw; nostril closer to snout than eye; loreal region oblique; canthus rostralis indistinct; eyes large (ED / HDL 0.40), eye diameter slightly longer than snout length (ED / SL 1.07), eyes notably protuberant in both dorsal and lateral views, pupil vertical; tympanum distinct, rounded, tympanum diameter smaller than eye (TYD / ED 0.38), upper margin of tympanum in contact with supratympanic ridge; vomerine teeth absent; tongue notched behind; supratympanic ridge distinct, extending from posterior corner of eye to supra-axillary gland.

Fore-limb relatively long (LAL / SVL 0.46), fingers long and slender (ML / SVL 0.25), webbing absent, lateral fringes on fingers narrow; relative finger lengths II < I < IV < III; tips of fingers rounded and slightly swollen; subarticular tubercles absent on fingers, inner metacarpal tubercle large and rounded, separated from the smaller, round outer metacarpal; supra-axillary glands oval.

Hind-limb relatively long (HLL / SVL 1.53), heels overlapping when the tibias perpendicular to the body axis; tibio-tarsal articulation of adpressed limb reaching middle of eye, tibia length about half of snout-vent length (TL / SVL 0.49); relative toe length: I < II < V < III < IV; toe tips rounded and slightly swollen; rudimentary webbing present between all five toes; wide lateral fringes present on all toes; dermal ridges under fourth toes interrupted; subarticular tubercles distinct under the base of II, III and IV toe; inner metatarsal tubercle oval and distinct, outer metatarsal tubercle absent (Fig. 3C).

Dorsal skin relatively smooth with small tubercles and short folds; supra-axillary gland distinct and yellowish; pectoral gland small and indistinct; round femoral glands present and protuberant on rear of thigh, closer to knee than to vent; femoral adipose glands distinct, attached to inner side of skin on posterior ventral surface of thigh; ventral skin smooth; ventrolateral glands forming a distinct white line on flanks.

##### Colouration of holotype in life.

In life, dorsal surface of head and trunk yellowish-brown, with distinct olive reverse-triangle dark markings between eyes connecting to a dark W-shaped marking between axillae that are fringed with greyish-white colour; elbow to upper arm distinctly yellowish-orange in colour on the dorsum; four transverse black bars present on dorsal surface of thighs and three on dorsal surface of lower arm; one dark blotch between nostril and eyes on loreal region and a dark blotch under the eye; supratympanic ridge reddish and a large black marking under supratympanic ridge; distinct dark blotches on ﬂanks from groin to axilla, longitudinally in two rows; ventral surfaces light coloured; throat and ventral arms pinkish with cream speckling on margins; chest and belly cream white, on the lateral belly with dense brown speckling; ventral hind-limbs pinkish with sparse white glands; upper iris copper, lower iris silver.

##### Preserved holotype colouration.

Dorsum of body and limbs fading to brown copper; transverse bars on limbs becoming more distinct. Ventral surface of body and limbs fading to cream white. Supra-axillary, femoral and pectoral glands fading to cream yellow.

##### Variations.

Measurements and basic statistics of adult specimens are presented in Suppl. materials 1, 3, respectively. Females larger than males (29.2–34.2 mm in 15 adult males and 34.4–43.1 mm in seven adult females) and, in CIB LB20220311002, dark blotch between nostril and eyes on loreal region absent (Fig. 5A); in CIBSCC1754, a patch on the outside of the reverse-triangle dark markings between eyes (Fig. 5B); in CIB LB20220305005, no longitudinal stripes along dorsolateral body (Fig. 5C); in CIB LB20220306008, the colouration of head and anterior dorsum is darker than the posterior (Fig. 5D).

##### Bioacoustics.

**Figure 6.**
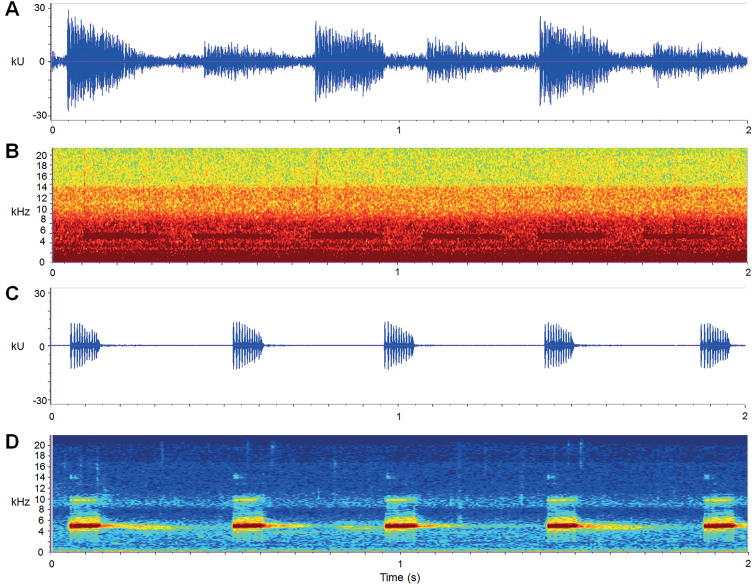
Advertisement calls of *Leptobrachelladong* sp. nov. **A, B** waveform and sonogram of the first call type (type A) over 2 seconds of the paratype CIB LB20220311002, respectively **C, D** waveform and sonogram of the second call type (type B) over 2 seconds of the holotype CIB SSC1757, respectively.

(Fig. 6; Suppl. material 3). Calls recorded at temperatures 11.2 to 15.0 °C. Descriptions based on six sequenced adults (Suppl. material 3) and 157 calls were measured. Dominant frequency of all type of calls is 4.4–5.6 kHz (5.1 ± 0.4) kHz, call duration is 203.783 ± 161.7 ms, call interval is 1238.3 ± 2034.5 ms, call repetition rate is 1.61 ± 0.9 (calls/s). Additionally, the calls were of two types. The first type (type A) consists of repeated short notes (Fig. 6A, B), and the second type (type B) consists of two repeated short notes and with longer call duration (524.2 ± 64.2 ms) and shorter call interval (148.8 ± 72.8 ms) than type A (Fig. 6C, D). Amplitude of type A was largest at first pulse, drastic reducing in the following pulses; amplitude of second note of type A about half of the first note; amplitude of type B with highest pulse at the beginning of each note and decreasing towards to the end.

##### Comparisons.

Compared with the 26 known congeners occurring south of the Isthmus of Kra, *Leptobrachelladong* sp. nov. could be distinguished from them by several characters: by having supra-axillary and ventrolateral glands, the new species differs from *L.arayai*, *L.dringi*, *L.fritinniens*, *L.gracilis*, *L.hamidi*, *L.heteropus*, *L.kajangensis*, *L.kecil*, *L.marmorata*, *L.maura*, *L.melanoleuca*, *L.picta*, *L.platycephala*, *L.sabahmontana*, and *L.sola* (vs. absent in the latter); by having rounded fingertips and moderate body size (29.2–34.2 mm in 15 adult males and 34.4–43.1 mm in seven adult females), the new species differs from the following species with pointed fingertips and smaller body size: *L.baluensis* (14.9–15.9 mm in males), *L.bondangensis* (17.8 mm in male), *L.brevicrus* (17.1–17.8 mm in males), *L.fusca* (16.3 mm in male), *L.itiokai* (15.2–16.7 mm in males), *L.juliandringi* (17.0–17.2 mm in males), *L.mjobergi* (15.7–19.0 mm in males), *L.natunae* (17.6 mm in one adult male), *L.palmata* (14.4–16.8 mm in males), *L.parva* (15.0–16.9 mm in males) and *L.serasanae* (16.9 mm in female).

*Leptobrachelladong* sp. nov. could also be identified from 65 known *Leptobrachella* species occurring north of the Isthmus of Kra by some characters (see Table 4).

**Table 3.** Basic statistics for the measurements of *Leptobrachelladong* sp. nov. Units in mm. See abbreviations for characters in the Materials and methods section.

Measurement	Male (n = 15)		Female (n = 7)	
	Ranging	Mean ± SD	Ranging	Mean ± SD
SVL	29.2–34.2	30.9 ± 1.4	34.4–43.1	39.4 ± 2.8
HDL	9.8–11.7	10.5 ± 0.6	11.8–13.8	12.7 ± 0.7
HDW	9.8–12.2	11.0 ± 0.7	12.4–14.5	13.7 ± 0.8
SL	4.0–5.0	4.5 ± 0.3	4.9–6.3	5.6 ± 0.4
IND	2.8–4.4	3.3 ± 0.4	3.4–4.7	4.3 ± 0.5
IOD	2.3–3.9	3.2 ± 0.5	3.4–4.1	3.6 ± 0.2
UEW	2.6–3.4	2.9 ± 0.2	2.9–4.3	3.6 ± 0.5
ED	3.6–4.4	4.1 ± 0.2	4.2–5.3	4.8 ± 0.4
TYD	1.5–2.2	1.7 ± 0.2	1.6–3.0	2.5 ± 0.4
LAL	13.5–15.5	14.6 ± 0.5	16.8–18.9	17.9 ± 0.9
ML	7.2–8.6	7.9 ± 0.4	8.6–10.5	9.6 ± 0.7
TL	14.5–16.4	15.2 ± 0.7	17.4–19.6	18.3 ± 0.8
FL	13.5–15.5	14.5 ± 0.6	17.4–19.4	17.9 ± 1.0
HLL	41.3–51.6	47.6 ± 3.1	54.6–62.5	58.4 ± 2.9

**Table 4.** Diagnosis characters on morphology of *Leptobrachelladong* sp. nov. from other congeners.

ID	Species	Male SVL (mm)	Female SVL (mm)	Black spots on flanks	Toes webbing	Fringes on toes	Ventral colouration	Dorsal skin texture
1	*Leptobrachelladong* sp. nov.	29.2–32.0	34.4–43.1	Yes	Rudimentary	Wide	White with distinct nebulous brown speckling on ventrolateral flanks	Shagreened with fine tubercles
2	*L.aerea*	25.1–28.9	27.1–38.6	No	Rudimentary	Wide	Near immaculate creamy-white, brown speckling on margins	Finely tuberculate
3	*L.alpina*	24.0–26.4	31.7–32.5	Yes	Rudimentary	Wide in males	Creamy-white with dark spots	Relatively smooth, some with small warts
4	*L.applebyi*	19.6–22.3	21.7	Yes	Rudimentary	No	Reddish-brown with white speckling	Smooth
5	*L.ardens*	21.3–24.7	25.4	Yes	No	No	Reddish-brown with white speckling	Smooth-finely shagreened
6	*L.aspera*	22.4	25.0–26.4	Yes	Rudimentary	Narrow	Creamy-white with distinct dark patches on chest and abdomen	Rough with dense conical granules, tubercles and glandular folds
7	*L.bashaensis*	22.9–25.6	27.1	Yes	Rudimentary	Narrow	Creamy-white chest and off-white belly with irregular black spots	Dorsal skin slightly shagreened with small tubercles and irregular brown stripes
8	*L.bidoupensis*	23.6–24.6	29.2–29.4	Yes	Rudimentary	Weak	Reddish-brown with white speckling	Smooth
9	*L.bijie*	29.0–30.4	/	Yes	Rudimentary	Narrow	White with distinct nebulous greyish speckling on chest and ventrolateral flanks	Shagreened and granular
10	*L.botsfordi*	29.1–32.6	30.0–31.8	No	Rudimentary	Narrow	Reddish-brown with white speckling	Shagreened
11	*L.bourreti*	27.4–36.2	39.5–45.0	Yes	Rudimentary	Weak	Creamy-white	Relatively smooth, some with small warts
12	*L.chishuiensis*	30.8–33.4	34.2	Yes	Rudimentary	Narrow	White with distinct nebulous greyish speckling on chest and ventrolateral ﬂanks	Shagreened and granular
13	*L.crocea*	22.2–27.3	/	No	Rudimentary	No	Bright orange	Highly tuberculate
14	*L.damingshanensis*	33.6–34.4	/	Yes	Rudimentary	Narrow	Creamy-white ventral surface with small, creamy-white glands on throat, chest and belly, becoming more concentrated near lateral margin	Rough dorsal skin with sparse jacinth tubercles and some short longitudinal ridges
15	*L.dorsospina*	28.7–30.5	32.1–39.8	Yes	Rudimentary	Narrow	Greyish-white with black spots and orange pigmentations	Rough with dense conical granules, tubercles, glandular folds and conical spines
16	*L.eos*	33.1–34.7	40.7	No	Rudimentary	Wide	Creamy-white	Shagreened
17	*L.feii*	21.5–22.8	25.7	Yes	Rudimentary	Narrow	Creamy-white with black blotches	Shagreened with small tubercles and ridge
18	*L.firthi*	26.4–29.2	25.7–36.9	No	Rudimentary	Wide in males	Creamy-white	Shagreened with fine tubercles
19	*L.flaviglandulosa*	23.0–27.0	29.3	Yes	Poorly developed	Narrow	Whitish, black speckling on margins	Shagreened with yellowish-brown tubercles
20	*L.fuliginosa*	28.2–30.0	/	Yes	Rudimentary	Weak	White with brown dusting	Nearly smooth, few tubercles
21	*L.graminicola*	23.1–24.6	28.6–32.9	No	Rudimentary	Wide	White with brown spots	Smooth, with many tubercles
22	*L.isos*	23.7–27.9	28.6–31.5	No	Rudimentary	Wide in males	Creamy-white with white dusting on margins	Mostly smooth, females more tuberculate
23	*L.jinshaensis*	29.7–31.2	/	Yes	No	Narrow	Cream yellow, presence of distinct nebulous greyish speckling on ﬂanks	Shagreened and granular
24	*L.kalonensis*	25.8–30.6	28.9–30.6	Yes	No	No	Pale, speckled brown	Smooth
25	*L.khasiorum*	24.5–27.3	31.2–33.4	Yes	Rudimentary	Wide	Creamy white	Isolated, scattered tubercles
26	*L.lateralis*	26.9–28.3	36.6	Yes	Rudimentary	No	Creamy white	Roughly granular
27	*L.laui*	24.8–26.7	28.1	Yes	Rudimentary	Wide	Creamy-white with dark brown dusting on margins	Round granular tubercles
28	*L.liui*	23.0–28.7	24.5–27.8	Yes	Rudimentary	Wide	Creamy-white with dark brown spots on chest and margins	Round granular tubercles with glandular folds
29	*L.macrops*	28.0–29.3	30.3	Yes	Rudimentary	No	Greyish-violet with white speckling	Roughly granular with larger tubercles
30	*L.maculosa*	24.2–26.6	27	Yes	No	No	Brown, less white speckling	Mostly smooth
31	*L.mangshanensis*	22.2–27.8	30.2	Yes	Rudimentary	Weak	White speckles on throat and belly	Nearly smooth
32	*L.maoershanensis*	25.2–30.4	29.1	Yes	Rudimentary	Narrow	Creamy-white chest and belly with irregular black spots	Longitudinal folds
33	*L.melica*	19.5–22.8	/	Yes	Rudimentary	No	Reddish-brown with white speckling	Smooth
34	*L.minima*	25.7–31.4	31.6–37.3	Yes	Rudimentary	No	Creamy-white	Smooth
35	*L.murphyi*	23.2–24.9	29.3–32.1	Yes	Rudimentary	Wide	Creamy-white belly with small black spots on the margin	Shagreened with reddish tubercles and folds
36	*L.nahangensis*	40.8	/	Yes	Rudimentary	No	Creamy-white with light specking on throat and chest	Smooth
37	*L.namdongensis*	30.9	32.1–35.3	Yes	Rudimentary	No	Creamy-white with brown dusting on margins	Finely tuberculate
38	*L.neangi*	/	35.4–36.3	Yes	Rudimentary (in females)	absent (in females)	Light purplish-grey with dark brown mottling on throat	Small, irregular bumps and ridges
39	*L.niveimontis*	22.5–23.6	28.5–28.7	Yes	Rudimentary	No	Ventral sides marbled with distinct irregular black speckling	Skin on dorsum scattered with fine reddish tubercles
40	*L.nokrekensis*	26.0–33.0	34.0–35.0	Yes	Rudimentary	unknown	White with distinct nebulous greyish speckling on chest and ventrolateral flanks	Tubercles and longitudinal folds
41	*L.nyx*	26.7–32.6	37.0–41.0	Yes	Rudimentary	No	Creamy-white with white and brown margins	Rounded tubercles
42	*L.oshanensis*	26.6–30.7	31.6	Yes	No	No	Whitish with no markings or only small, light grey spots	Smooth with few glandular ridges
43	*L.pallida*	24.5–27.7	/	No	No	No	Reddish-brown with white speckling	Tuberculate
44	*L.pelodytoides*	27.5–32.3	/	Yes	Wide	Narrow	Whitish	Small, smooth warts
45	*L.petrops*	23.6–27.6	30.3–47.0	No	No	Narrow	Immaculate creamy white	Highly tuberculate
46	*L.pluvialis*	21.3–22.3	25.5–33.5	Yes	Rudimentary	No	Dirty white with dark brown marbling	Smooth, flattened tubercles on flanks
47	*L.puhoatensis*	24.2–28.1	27.3–31.5	Yes	Rudimentary	Narrow	Reddish-brown with white dusting	Longitudinal skin ridges
48	*L.purpurus*	25.0–27.5	/	Yes	Rudimentary	Wide	Dull white with indistinct grey dusting	Shagreen with small tubercles
49	*L.purpuraventra*	27.3–29.8	33.0–35.3	Yes	Rudimentary	Narrow	Grey purple with distinct nebulous greyish speckling on chest and ventrolateral flanks	Shagreened and granular
50	*L.pyrrhops*	30.3–33.9	30.8–34.3	Yes	Rudimentary	No	Reddish-brown with white speckling	Slightly shagreened
51	*L.rowleyae*	23.4–25.4	27.0–27.8	Yes	No	No	Pinkish milk-white to light brown chest and belly with numerous white speckles	Smooth with numerous tiny tubercles
52	*L.shangsiensis*	24.9–29.4	30.8–35.9	Yes	Rudimentary	Narrow	ventral surface yellowish-creamy-white with marble texture	Smooth
53	*L.shiwandashanensis*	26.8–29.7	/	Yes	No	No	Creamy-white ventral surface with small, creamy-white glands on throat, chest and belly, becoming more concentrated near lateral margin	Shagreened with small raised tubercles and ridges
54	*L.sungi*	48.3–52.7	56.7–58.9	No or small	Wide	Weak	White	Granular
55	*L.suiyangensis*	28.7–29.7	30.5–33.5	Yes	Rudimentary	Narrow	Yellowish-creamy-white with marble texture chest and belly or with irregular light brown speckling	Shagreen with small granules
56	*L.tadungensis*	23.3–28.2	32.1	Yes	No	No	Reddish-brown with white speckling	Smooth
57	*L.tamdil*	32.3	32.3	Yes	Wide	Wide	White	Weakly tuberculate
58	*L.tengchongensis*	23.9–26.0	28.8–28.9	Yes	Rudimentary	Narrow	White with dark brown blotches	Shagreened with small tubercles
59	*L.tuberosa*	24.4–29.5	30.2	No	Rudimentary	No	White with small grey spots/streaks	Highly tuberculate
60	*L.ventripunctata*	23.7–27.7	31.5–35.0	Yes	Rudimentary	No	Chest and belly with dark brown spots	Longitudinal skin ridges
61	*L.wuhuangmontis*	25.6–30.0	33.0–36.0	Yes	Rudimentary	Narrow	Greyish-white mixed by tiny white and black dots	Rough, scattered with dense conical tubercles
62	*L.wulingensis*	24.5–32.8	29.9–38.5	Yes	Rudimentary	Narrow	Creamy white, with distinct or indistinct brown speckling at margins	Shagreened with sparse large warts, sometimes with longitudinal ridges
63	*L.yeae*	25.8–32.6	33.7–34.1	Yes	Rudimentary	Narrow	Ventral belly cream white with variable brown specking	Dorsum relatively smooth with fine tiny granules or short ridges
64	*L.yingjiangensis*	25.7–27.6	/	Yes	Rudimentary	Wide	Creamy-white with dark brown flecks on chest and margins	Shagreened with small tubercles
65	*L.yunkaiensis*	25.9–29.3	34.0–35.3	Yes	Rudimentary	Wide	Belly pink with distinct or indistinct speckling	Shagreened with short skin ridges and raised warts
66	*L.zhangyapingi*	45.8–52.5	/	No	Rudimentary	Wide	Creamy-white with white and brown	Mostly smooth with distinct tubercles
67	*L.pingbianensis*	28	30	Yes	Rudimentary	Unknown	Chest and belly with dark brown spots	Smooth
68	*L.shimentaina*	26.4–8.9	30.1–30.7	Yes	Rudimentary	Wide in males	Greyish-pink with distinct hazy brown speckling on chest and ventrolateral flanks	Round granular tubercles with glandular folds
69	*L.verrucosa*	23.2–25.9	/	Yes	Rudimentary	Narrow	Creamy-white with greyish-white and dark brown spots	Shagreened with numerous conical tubercles
70	*L.yunyangensis*	28.3–30.6	/	Yes	Rudimentary	Narrow	Light greyish-creamy-white, interspersed with light brown spots	Rough with sparse large warts, with short longitudinal ridges

By having medium size of body (SVL 29.2–34.2 mm in males) *Leptobrachelladong* sp. nov. differs from the smaller males *L.aerea* (25.1–28.9 mm), *L.alpina* (24.0–26.4 mm), *L.applebyi* (19.6–22.3 mm), *L.ardens* (21.3–24.7 mm), *L.aspera* (22.4 mm), *L.bashaensis* (22.9–25.6 mm), *L.bidoupensis* (23.6–24.6), *L.crocea* (22.2–27.3 mm), *L.feii* (21.5–22.8 mm), *L.flaviglandulosa* (23.0–27.0 mm), *L.isos* (23.7–27.9 mm), *L.graminicola* (23.1–24.6 mm), *L.khasiorum* (24.5–27.3 mm), *L.lateralis* (26.9–28.3 mm), *L.laui* (24.8–26.7 mm), *L.liui* (24.8–26.7 mm), *L.maculosa* (24.2–26.6 mm), *L.mangshanensis* (22.22–27.76 mm), *L.maura* (26.1 mm), *L.melica* (19.5–22.8 mm), *L.murphyi* (23.2–24.9 mm), *L.niveimontis* (22.5–23.6 mm), *L.pallida* (24.5–27.7 mm), *L.petrops* (23.6–27.6 mm), *L.pluvialis* (21.3–22.3 mm), *L.puhoatensis* (24.2–28.1 mm), *L.pyrrhops* (25.0–27.5 mm), *L.rowleyae* (23.4–25.4 mm), *L.tadungensis* (23.3–28.2 mm), *L.tengchongensis* (23.9–26.0 mm), *L.ventripunctata* (23.7–27.7 mm) and *L.yingjiangensis* (25.7–27.6 mm) and differs from the larger in males *L.nahangensis* (40.8 mm), *L.platycephala* (35.1 mm), *L.sungi* (48.3–52.7 mm in males) and *L.zhangyapingi* (45.8–52.5 mm).

By having a larger size of body (SVL 34.4–43.1 mm in females), *Leptobrachelladong* sp. nov. differs from the smaller females *L.alpina* (31.7–32.5 mm), *L.applebyi* (21.7 mm), *L.ardens* (25.4 mm), *L.aspera* (25.0–26.4 mm), *L.bashaensis* (27.1), *L.botsfordi* (30.0–31.8 mm), *L.graminicola* (28.6–32.9 mm), *L.isos* (28.6–31.5 mm), *L.kalonensis* (28.9–30.6 mm), *L.khasiorum* (31.2–33.4 mm), *L.liui* (24.5–27.8 mm), *L.macrops* (30.3 mm), *L.maculosa* (27 mm), *L.mangshanensis* (30.2 mm), *L.maoershanensis* (29.1 mm), *L.murphyi* (29.3–32.1 mm), *L.macrops* (30.3 mm), *L.maoershanensis* (29.1 mm), *L.niveimontis* (28.5–28.7 mm), *L.oshanensis* (31.6 mm), *L.pluvialis* (25.5–33.5 mm), *L.puhoatensis* (27.3–31.5 mm), *L.rowleyae* (27.0–27.8 mm), *L.shimentaina* (30.1–30.7 mm), *L.suiyangensis* (30.5–33.5 mm), *L.tadungensis* (32.1 mm), *L.tamdil* (32.3 mm), *L.tengchongensis* (28.8–28.9 mm) and *L.tuberosa* (30.2 mm).

By having black spots on ﬂanks, *Leptobrachelladong* sp. nov. differs from *L.aerea*, *L.botsfordi*, *L.crocea*, *L.eos*, *L.firthi*, *L.isos*, *L.pallida*, *L.petrops*, *L.tuberosa* and *L.zhangyapingi* (vs. lacking distinct black spots on the ﬂanks in the latter).

By having rudimentary webbing, *Leptobrachelladong* sp. nov. differs from *L.ardens*, *L.jinshaensis*, *L.kalonensis*, *L.maculosa*, *L.oshanensis*, *L.pallida*, *L.petrops*, *L.rowleyae*, *L.shiwandashanensis* and *L.tadungensis* (vs. absent webbing in the latter).

By having wide fringes on toes, *Leptobrachelladong* sp. nov. differs from *L.applebyi*, *L.ardens*, *L.aspera*, *L.bashaensis*, *L.bidoupensis*, *L.bijie*, *L.botsfordi*, *L.bourreti*, *L.chishuiensis*, *L.crocea*, *L.damingshanensis*, *L.dorsospina*, *L.feii*, *L.flaviglandulosa*, *L.fuliginosa*, *L.jinshaensis*, *L.kalonensis*, *L.lateralis*, *L.macrops*, *L.maculosa*, *L.mangshanensis*, *L.melica*, *L.minima*, *L.nahangensis*, *L.namdongensis*, *L.niveimontis*, *L.nyx*, *L.oshanensis*, *L.pallida*, *L.pelodytoides*, *L.petrops*, *L.pluvialis*, *L.puhoatensis*, *L.purpuraventra*, *L.pyrrhops*, *L.rowleyae*, *L.shangsiensis*, *L.shiwandashanensis*, *L.sungi*, *L.tengchongensis*, *L.tuberosa*, *L.ventripunctata*, *L.verrucosa*, *L.wuhuangmontis*, *L.wulingensis*, *L.yeae* and *L.yunyangensis* (vs. fringes on toes narrow or absent in the latter).

By having dorsal surface shagreened with fine tubercles, *Leptobrachelladong* sp. nov. differs from *L.applebyi*, *L.bidoupensis*, *L.kalonensis*, *L.melica*, *L.minima*, *L.nahangensis*, *L.pingbianensis*, *L.shangsiensis* and *L.tadungensis*, all of which have the dorsum smooth and *L.bourreti* (dorsum smooth with small warts), *L.fuliginosa* (dorsum smooth with fine tubercles), *L.liui* (dorsum with round tubercles), *L.macrops* (dorsum roughly granular with large tubercles), *L.maoershanensis* (dorsum shagreened with tubercles), *L.minima* (dorsum smooth), *L.neangi* (dorsum with small, irregular bumps and ridges), *L.nyx* (dorsum with round tubercles), *L.nokrekensis* (dorsum tubercles and longitudinal folds), *L.pelodytoides* (dorsum with small, smooth warts), *L.tamdil* (dorsum weakly tuberculate, with low, oval tubercles), *L.tuberosa* (dorsum highly tuberculate), *L.yunkaiensis* (dorsum with raised warts) and *L.wuhuangmontis* (dorsum rough with conical tubercles).

The advertisement calls of *Leptobrachelladong* sp. nov. (Results and Fig. 3) differs from all other congeners occurring north of the Isthmus of Kra for which comparable acoustic data are available consisting of uniform and continuous calls with four to five pulses in each note. Of the congeners in the region with known calls, the new species can be separated from *L.purpurus*, *L.tuberosus*, *L.puhoatensis* and *L.yingjiangensis* by not having an invariably single-note call with irregular intervals. In addition, the dominant frequency of 4.4–5.6 kHz (at 11.2–15 °C) further distinguishes the call of *Leptobrachelladong* sp. nov. from that of the higher frequency calls of *L.aereus* (6.2–7.9 kHz at 22.4–25.7 °C), *L.yingjiangensis* (5.7–5.9 kHz at 19 °C), *L.isos* (5.9–6.2 kHz at 22.4–22.8 °C) and *L.ventripunctatus* (6.1–6.4 kHz at 15 °C) and the lower frequency calls of *L.applebyi* (4.0–4.3 kHz at 21.5 °C), *L.ardens* (3.1–3.4 kHz at 21.4–24.7 °C), *L.bidoupensis* (1.9–3.8 kHz at 19–21 °C), *L.botsfordi* (2.6–3.2 kHz at 14 °C), *L.croceus* (2.6–3.0 kHz at 21.6–25.1 °C), *L.fuliginosus* (2.1–2.8 kHz at 19.3–19.6 °C), *L.kalonensis* (2.8 kHz at 26.4 °C), *L.maculosus* (2.7–2.8 kHz at 23.3–24.1 °C), *L.melicus* (2.6–4.0 kHz at 26.1–26.2 °C), *L.pallidus* (2.4–2.7 kHz at 14.0–21.4 °C), *L.pyrrhops* (1.91–2.2 kHz at 25 °C), *L.rowleyae* (3.3–3.5 kHz at 21.5 °C), *L.tadungensis* (2.6–3.1 kHz at 12.9–22.3 °C) and *L.tuberosus* (2.6–2.8 kHz at 22.5–24.5 °C).

In mitochondrial DNA trees, *Leptobrachelladong* sp. nov. was clustered as an independent clade and sister to a clade comprising of *L.graminicola* and *L.yeae*. The new species differs from *L.graminicola* by the following characters: body size larger with SVL 29.2–34.2 mm in adult males and 34.4–43.1 mm in adult females (vs. 23.1–24.6 mm in adult males and 28.6–32.9 mm in adult females); black spots on flanks present (vs. absent); ventral surface white with distinct nebulous brown speckling on ventrolateral flanks (vs. white with brown spots); dorsal surface shagreened with fine tubercles (vs. smooth, with many tubercles); and tibiotarsal articulation reaching to middle of eye (vs. anterior edge of eye). The new species differs from *L.yeae* by having wide fringes on toes (vs. narrow); dorsal surface shagreened with fine tubercles (vs. relatively smooth with fine tiny granules or short ridges); and males with a pair of subgular internal vocal sacs (vs. internal single subgular vocal sac).

##### Tadpoles

**Figure 7.**
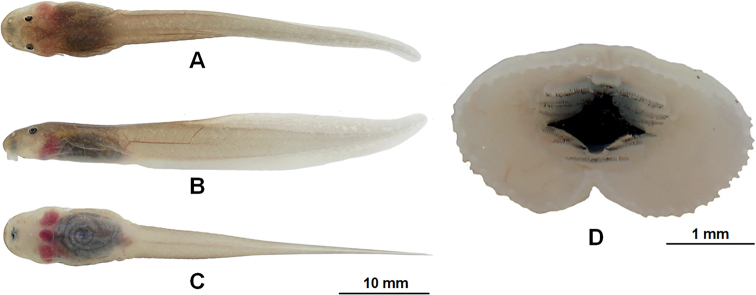
The tadpole CIB WB2020277 of *Leptobrachelladong* sp. nov. in life **A** dorsal view **B** lateral view **C** ventral view **D** oral disc.

**(in mm).** Description based on sequenced tadpole CIB WB2020277 at Gosner stage 27 (Fig. 7). Body elliptical elongate in dorsal view; slightly depressed (BH / BW 1.5, BH 4.7, BL 14.7); eyes lateral (ED 0.9), nostril near to snout than eye (NE 2.9, RN 1.6, IND 2.4, SN 3.5); spiracle on left side of body (SS 7.8); keratodont formula I: 3+3/2+2: I; oral disc is cup-shaped with labial papillae (ODW 3.4); TOL 65.8 mm; tail fusiform, approximately 2.5 times as long as snout-vent length, tail height 18.2% of tail length (TH 6.4, TMH 4.6, TMW 3.8); dorsal fin low, arising behind the origin of the tail (SU 20.1); maximum tail depth near mid-length of tail and larger than body depth (TH / BL 1.4, UF 2.1, LF 1.3); the tip of tail rounded and without spots on dorsal of body.

##### Secondary sexual characteristics.

**Figure 8.**
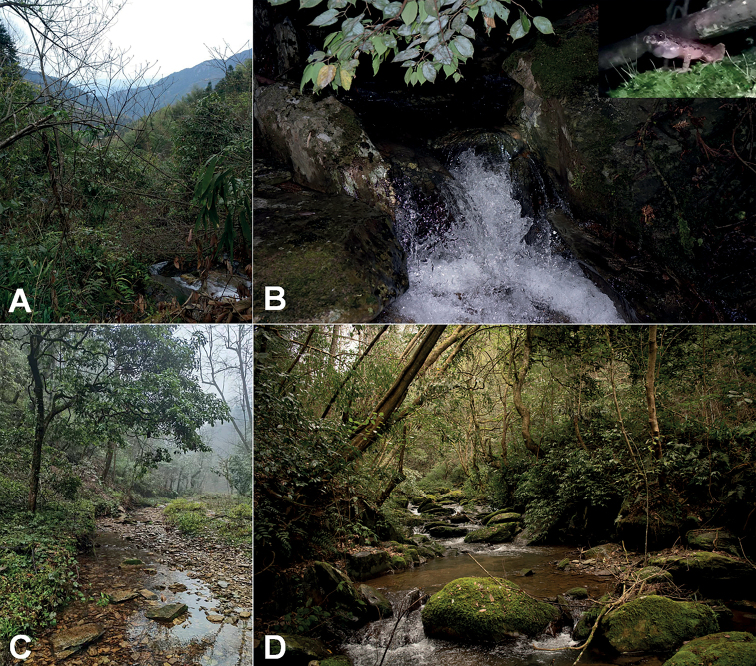
Habitats of *Leptobrachelladong* sp. nov. **A** landscape of the type locality Tongdao County, Hunan Province, China **B** a torrent mountain stream in the type locality (insert holotype CIB SSC1757 in life in the field) **C** habitat at the Congjiang County, Guizhou Province, China **D** habitat at the Suining County, Hunan Province, China.

Adult males with a pair of subgular vocal sacs (Fig. 8B), femoral adipose glands present on posterior surface of thigh and tiny transparent spines on chest during breeding season. Nuptial pads and spines absent on males.

##### Ecology notes.

*Leptobrachelladong* sp. nov. has been found in three localities: Tongdao County and Suining County, Hunan Province and Congjiang County, Guizhou Province, China. Elevations recorded range from 620 m to 1200 m. Population from the Tongdao County inhabited a torrent stream covered by evergreen shrubs and the new species always found on the stones (Fig. 8A, B). Population from Congjiang County inhabited slow-flowing streams surrounded by evergreen broadleaf forest (Fig. 8C). Populations from Suining County, Hunan Province inhabited broad mountain stream surrounded by evergreen broadleaf forest (Fig. 8D). Tadpoles could be found at daytime and night. Gravid females were found by the streams in the type locality (2 April 2017) and Suining County (15 March 2022).

##### Etymology.

This specific name “dong” refers to the Dong people, as the new species distributed in the concentrated area of Dong people. We suggest its English common name “Dong leaf litter toads” and Chinese name “Dong Zhang Tu Chan (侗掌突蟾)”.

## ﻿Discussion

South-western China was proposed as a biodiversity hotspot (Myers et al. 2000). In the past five years, 34 new species of the genus *Leptobrachella* have been discovered (Frost 2022), while the species of *Leptobrachella* have low vagility and an exclusive association with montane forests and their populations are often highly structured and underestimation of species diversity occurs in the genus, which suggests a high degree of localised diversification and micro-endemism (Fei et al. 2012; Chen et al. 2018). Therefore, a lot of cryptic species were proposed by molecular analyses in areas where surveys are weak (Chen et al. 2018).

This new species was found in three localities and the largest geographical distance between the localities is over 200 km. However, in this study, phylogenetic analyses, based on mitochondrial DNA, suggested the three populations as the same species and different from its congeners on a series of morphological characters. This perhaps indicated that the species have a widespread distribution. Further surveys are needed to evaluate the population status of the species.

## Supplementary Material

XML Treatment for Leptobrachella dong

## Supplementary materials

Supplementary material 1 Measurements of adult specimen of *Leptobrachelladong* sp. nov. Data type table (excel document) Explanation note Morphological data. Units in mm. See abbreviations for characters in the Materials and methods section. https://binary.pensoft.net/file/814360This dataset is made available under the Open Database License (http://opendatacommons.org/licenses/odbl/1.0/). The Open Database License (ODbL) is a license agreement intended to allow users to freely share, modify, and use this Dataset while maintaining this same freedom for others, provided that the original source and author(s) are credited.Author: Bin Wang

Supplementary material 2 Uncorrected p-distance between *Leptobrachella* species on the 16S rRNA gene Data type table (excel document) Explanation note Genetic distance. The values below 3% are coloured in red. https://binary.pensoft.net/file/814361This dataset is made available under the Open Database License (http://opendatacommons.org/licenses/odbl/1.0/). The Open Database License (ODbL) is a license agreement intended to allow users to freely share, modify, and use this Dataset while maintaining this same freedom for others, provided that the original source and author(s) are credited.Author: Bin Wang

Supplementary material 3 Call data Data type table (excel document) Explanation note Call measurements (mean ± standard deviation) of *Leptobrachelladong* sp. nov. https://binary.pensoft.net/file/814362This dataset is made available under the Open Database License (http://opendatacommons.org/licenses/odbl/1.0/). The Open Database License (ODbL) is a license agreement intended to allow users to freely share, modify, and use this Dataset while maintaining this same freedom for others, provided that the original source and author(s) are credited.Author: Bin Wang
